# Predictive biomarkers for cardiometabolic risk in postmenopausal women: insights into visfatin, adropin, and adiponectin

**DOI:** 10.3389/fendo.2025.1527567

**Published:** 2025-02-07

**Authors:** Anna Maria Cybulska, Daria Schneider-Matyka, Ireneusz Walaszek, Mariusz Panczyk, Dorota Ćwiek, Anna Lubkowska, Elżbieta Grochans, Kamila Rachubińska, Katarzyna Malewicz, Mariusz Chabowski

**Affiliations:** ^1^ Department of Nursing, Pomeranian Medical University in Szczecin, Szczecin, Poland; ^2^ Department of Education and Research in Health Sciences, Faculty of Health Sciences, Medical University of Warsaw, Warsaw, Poland; ^3^ Department of Obstetrics and Pathology of Pregnancy, Pomeranian Medical University in Szczecin, Szczecin, Poland; ^4^ Department of Functional Diagnostics and Physical Medicine, Pomeranian Medical University in Szczecin, Szczecin, Poland; ^5^ Geriatrics and Long-Term Care Department, Department of Nursing, Faculty of Nursing and Midwifery, Wroclaw Medical University, Wroclaw, Poland; ^6^ Department of Surgery, 4th Military Clinical Hospital, Wroclaw, Poland; ^7^ Department of Clinical Surgical Sciences, Faculty of Medicine, Wroclaw University of Science and Technology, Wroclaw, Poland

**Keywords:** adiponectin, adropin, BMI, menopausal women, obesity, visfatin

## Abstract

**Background:**

Visfatin, adropin, and adiponectin are involved in many changes associated with obesity and metabolic disorders, and may be related to metabolic syndrome and cardiovascular disease. The selection of visfatin, adropin, and adiponectin as biomarkers is based on their significant roles in metabolic regulation and inflammation, which are critical factors in cardiometabolic risk. Visfatin is known for its pro-inflammatory properties and its ability to modulate insulin resistance. Adropin is involved in energy homeostasis and metabolic health, while adiponectin has anti-inflammatory and insulin-sensitizing effects. During the perimenopausal period, the risk of obesity, and consequently cardiometabolic diseases increases. Therefore, the aim of this study was to assess the relationship between cardiometabolic parameters and circulating levels of visfatin, adropin, and adiponectin in perimenopausal women with regard to their obesity status.

**Materials and methods:**

This study of 168 perimenopausal women utilized a cross-sectional design with non-random sampling. It involved the use of questionnaires, as well as anthropometric and blood pressure measurements. Blood samples were collected to determine the levels of visfatin, adropin, and adiponectin. Statistical analyses, including correlation coefficients, were performed to evaluate the relationship between these biomarkers and cardiometabolic risk factors, such as insulin resistance, lipid profiles, and inflammatory markers.

**Results:**

In our study, visceral adiposity index and lipid accumulation product negatively correlated with adiponectin levels. Preliminary multivariate linear regression analysis revealed a positive correlation between circulating visfatin and IL-6 levels. Circulating adropin negatively correlated with HbA1C, fasting blood glucose, and insulin. Adiponectin negatively correlated with HbA1C, fasting blood glucose, insulin, and triglycerides. Furthermore, circulating adiponectin positively correlated with HDL, and negatively with HOMA-IR.

**Conclusions:**

Adiponectin is a promising biomarker for predicting cardiometabolic risk in postmenopausal women.

## 1 Introduction

The World Health Organization (WHO) has described natural menopause as the permanent cessation of menstrual bleeding resulting from the loss of ovarian follicular activity (1). With menopause, the risk of obesity and the amount of abdominal and visceral fat increase, further exacerbating the associated cardiometabolic risk. Obesity has serious consequences, such as an increased risk of cardiovascular disease, non-alcoholic fatty liver disease, metabolic syndrome (MetS), type 2 diabetes mellitus (T2DM), and several types of cancer (2–4). Obesity results from a combination of behavioral, epigenetic, genetic, physiological, socio-cultural, and environmental factors, which lead to an imbalance between energy intake and expenditure (5).

Abdominal fat can produce various autocrine, endocrine, and paracrine agents that impact the cardiovascular system and metabolism (6–9). Several methods exist to assess body fat mass and distribution, such as dual-energy X-ray absorptiometry (DXA), hydrostatic weighing, bioelectrical impedance analysis (BIA), and skinfold thickness measurement (10). However, these methods are limited by cost and complexity. Epidemiological studies suggest that simple anthropometric measurements, such as body mass index (BMI), waist-hip ratio (WHR), waist circumference (WC), waist-to-height ratio (WHtR), and body adiposity index (BAI) are effective indicators of obesity and its association with metabolic disorders and cardiovascular disease (11–13). Despite BMI’s role as a general obesity indicator, it does not fully reflect metabolic status or fat distribution due to the “obesity paradox” (13). Newer measures, including body shape index (BSI), lipid accumulation product (LAP), and body roundness index (BRI), are more predictive of obesity status and MetS risk (14, 15). BSI evaluates the health impact of height, weight, and WC, while BRI predicts body and visceral fat percentage using WC relative to height (16, 17). Visceral adipose tissue (VAT), a metabolically active tissue, is linked to metabolic disorders, inflammation, and impaired lipid levels. Excess VAT, not overall obesity, drives cardiometabolic risk and related mortality. In menopausal women, WHtR, WHR, BRI, and LAP are particularly useful for predicting cardiovascular disease (CVD) risk. A review of the literature does not clarify which obesity index is the best predictor of cardiometabolic diseases in perimenopausal women. However, as some studies show, BSI and BRI are better predictors of diabetes and premature death for cardiometabolic and obesity reasons than BMI and WC (18, 19).

Adropin, first described in 2008 as a secreted peptide consisting of 76 amino acids, plays a significant role in regulating energy metabolism and endothelial function. It is suggested to be involved in endothelial dysfunction, insulin resistance (IR), and energy homeostasis (20–23). According to the literature, serum adropin levels are influenced by diet and metabolic diseases. Numerous studies indicate an inverse correlation between adropin levels and BMI (24–30), where low levels are characteristic of obese individuals. Moreover, adropin levels can be affected by age, sex, cholesterol levels, and diabetes (31–33). A growing body of evidence highlights adropin’s role in modulating cardiovascular function, with altered tissue levels observed in various physiological and pathological conditions such as multiple sclerosis, COVID-19, gestational diabetes, rheumatoid arthritis, acute mesenteric ischemia, and diabetic nephropathy (34–39).

Visfatin (VISF) is one of the most important adipokines secreted by adipose tissue. It plays an important role in immunity, metabolism, aging, inflammation, and stress responses. Visfatin, which can be found in two forms (extracellular and intracellular), performs many functions. It modulates pathophysiological processes associated with obesity and MetS (such as inflammation and angiogenesis), and is involved in related diseases, including diabetes, cardiovascular complications, and some cancers. In addition, visfatin induces the secretion of pro- and anti-inflammatory cytokines (IL-1β, IL-6, IL-1Ra, IL-10 and TNF-α) in monocytes, participates in the cellular energy system and in the differentiation of fat cells, and induces triglyceride accumulation in preadipocytes and accelerates triglyceride synthesis from glucose (40).

Adiponectin (ADPN) is a monomeric protein, synthesized and secreted in white adipose tissue (41). It has been observed that adiponectin levels inversely correlate with the amount of body fat, so the highest levels of adiponectin are observed in under-weight and normal-weight individuals (42). Adiponectin regulates glucose metabolism (increases glucose uptake by tissues, reduces gluconeogenesis), thus protecting β-cells (43). It also defends the cardiovascular system by protecting myocardial cells, improving endothelial cell function, reducing oxidative stress and inflammation, and slowing down the development of cardiovascular disease. Adiponectin has a pleiotropic effect: it acts against diabetes (as it increases organ sensitivity to insulin), inflammation, atherosclerosis, and cancers (44).

Menopause introduces profound hormonal changes that significantly impact the levels and functions of various adipokines, including visfatin, adropin, and adiponectin. The decline in estrogen levels during menopause is a key factor associated with increased inflammation, insulin resistance, and elevated cardiovascular risk. Understanding the intricate roles of visfatin, adropin, and adiponectin during menopause is crucial for developing targeted interventions aimed at improving cardiometabolic health. These adipokines serve as valuable biomarkers for predicting and managing the heightened risk of cardiovascular diseases and metabolic disorders associated with menopause (20, 25, 40, 43, 44).

Many studies have reported that body fat mass increases in aging women, but the extent to which menopausal status mediates these changes remains unclear. The aim of this study was to assess how cardiometabolic parameters are linked to the levels of circulating visfatin, adropin, and adiponectin in perimenopausal women depending on their obesity status.

## 2 Materials and methods

### 2.1 Study design

#### 2.1.1 Participant *s*election

The study involved 205 perimenopausal women living in the West Pomeranian Voivodeship. It was approved by the Bioethics Committee of the Pomeranian Medical University in Szczecin (KB-0012/181/13), and was conducted in accordance with the Declaration of Helsinki. It was a cross-sectional study based on non-random sampling. Recruitment for the study was done via information posters in public places and advertisements in local newspapers. The size of the study sample was determined based on statistical data concerning the population of 45–64 year old females in the West Pomeranian Voivodeship in 2022 (45). The confidence level was 95%, the maximum error at 7%, and the estimated fraction size at 0.5.

- Subjects who met the following inclusion criteria were included in the study:
- female sex,
- the age of 45–64 years,
- normal mammography results,
- normal cervical smear results,
- no clinically confirmed mental disease,
- no clinically confirmed metabolic disease (stroke, cancer, vascular disease, renal or liver failure, thyroid disease, autoimmune disease, etc.),
- not taking menopausal hormone therapy (MHT),
- having a normal diet based on Polish cuisine, without supplementation of fatty acids (including omega-3),
- being a Caucasian woman living in the West Pomeranian Voivodeship in Poland,
- informed written consent to participate in the study.

**Figure 1:**
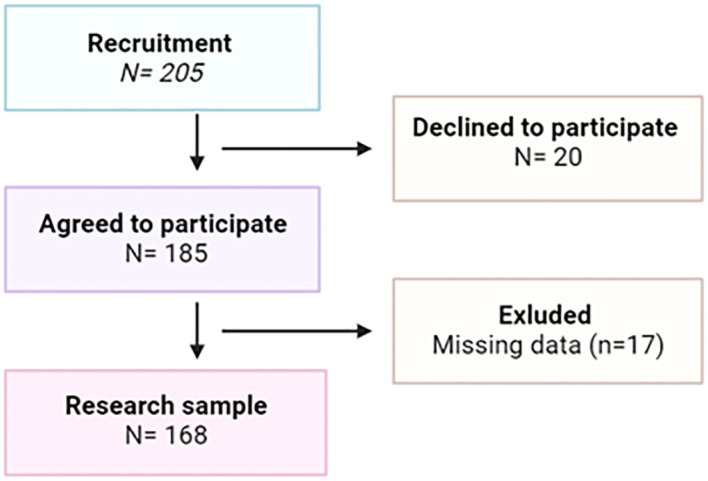
The study flow chart.

The exclusion criteria were current thyroid, neoplastic, and mental diseases, as well as history of these diseases. Ultimately, 168 respondents who met all the inclusion criteria were involved in the study (completion rate: 82%). This study is part of a larger project to assess the health status of perimenopausal women from the West Pomeranian Voivodeship (**Figure 1**).

#### 2.1.2 Data collection instruments

This survey-based study was carried out in several stages, namely completion of a questionnaire, anthropometric measurements, blood pressure measurements, and laboratory analysis.

The data collection procedure involved filling out a questionnaire to collect basic information on sociodemographic and health data. The participants provided information on their education, place of residence, marital status, employment status, menopausal status, as well as the use of stimulants and medications.

#### 2.1.3 Anthropometric measurements

Anthropometric measurements were collected in keeping with the recommendations of the International Society for the Advancement of Kinanthropometry (ISAK) (46). The following were measured:

- Weight and height—measured using a legal-for-trade medical scale with an integrated SECA 711 height meter, according to a standardized procedure with an accuracy of 0.1 kg and 0.1 cm, respectively. The respondents stood with their backs straight, heels together, barefoot, in light clothing.
- Body mass index (BMI)—calculated according to the formula: BMI = weight [kg]/height [m]^2^. As proposed by The Centers for Disease Control and Prevention (CDC), BMI was divided into the following categories: underweight (BMI < 18.5), normal weight (BMI = 18.5–24.9), overweight (BMI = 25.0–29.9), and obesity (BMI ≥ 30) (47).
- Waist circumference (WC)—measured to the nearest 0.01 m using an elastic measuring tape (SECA 711). WC was measured as the horizontal distance around the abdomen at the level of the navel. Abdominal obesity was defined as WC ≥ 80 cm (for European women) (48).
- Waist to height ratio (WHtR)—calculated according to the formula: WHtR = WC [cm]/height [cm]) (48).
- Relative fat mass (RFM)—calculated according to the formula: RFM = 76 – (20 x (height [m]/WC [m]). The result of the RFM equation is written in percent. It reflects the approximate percentage of body fat in the human body. For obese women, the percentage of body fat is ≥ 32% (49).
- Visceral adiposity index (VAI)—calculated according to the formula: VAI = [WC/39.68 + (1.88 × BMI)] × (TG/1.03) × (1.31/HDL). This indicator helps to assess visceral adipose dysfunction and cardiometabolic risk (49),
- Lipid accumulation product (LAP) for women—calculated according to the formula: WC (cm) − 58 * TG (mmol/l) (50).
- Body roundness index (BRI) (51)—calculated according to the formula: BRI = 364.2 − 365.5 × √(1 − (((WC/2π)^2^)/[(0.5 × height)]^2^)).
- Body shape index (BSI) (52)—calculated according to the formula: BSI = WC/[(BMI)^2/3^ × (height)^1/2^.

Blood pressure (BP) was measured using the Korotkov method in accordance with the recommendations of the American Heart Association (53). We took care to ensure proper positioning of the patient, a period of quiet rest, the use of an appropriately sized cuff, and the minimization of external factors that affect blood pressure, such as smoking and taking caffeinated products before blood pressure measurement (54).

#### 2.1.4 Biochemical measurements

Blood was collected from each respondent after an overnight fast, between 7:00 a.m. and 9:30 a.m. after a 10-minute rest in a sitting position. Blood was collected from the ulnar vein using the Vacutainer system by qualified nurses following applicable rules and procedures for the collection, storage and transport of biological material. The levels of biochemical parameters were determined in a certified laboratory of the Pomeranian Medical University in Szczecin by standardized commercial methods. Blood was collected to assess the levels of: insulin, glucose, glycated hemoglobin (HbA1C), total cholesterol (TC), high-density lipoprotein (HDL), low-density lipoprotein (LDL), triglycerides (TG), and C-reactive protein (CRP). Fasting blood glucose (FBG) and fasting insulin levels were used to calculate the homeostasis model assessment of insulin resistance (HOMA-IR) according to the formula (55): HOMA-IR = insulin (mU/ml) x glucose (mmol/l)/22.5. Additionally, plasma levels of adiponectin, visfatin, and adropin were determined using commercially available quantitative assay kits from the R&D System (cat. no. DLP000, cat. no. DRP300, Minneapolis, MN, USA, respectively). The Bio-Rad iMark™ Microplate Absorbance Reader was used for the biochemical analysis. Blood for determination of selected serum inflammatory markers was collected into serum separation tubes. Serum IL-1α, IL-1β, IL-6, TNFα, and IFNγ levels were measured by immunoassay using ELISA kits (DRG, Germany). The sensitivity of the IL-1α assay was 1.1 pg/ml, the intra- and interassay CV values were < 5.4% and < 10%, respectively. The sensitivity of the IL-6 assay was 2 pg/ml, the intra- and interassay CV values were 4.2% and 4.4%, respectively. The sensitivity of IL-1β assay was 0.35 pg/ml, the intra- and interassay CV values were 2.3% and 4.9%, respectively. The sensitivity of IFNγ assay was 0.03 IU/ml, the intra- and interassay CV values were 3.2% and 5.8%, respectively. The sensitivity of TNFα assay was 0.7 pg/ml.

### 2.2 Cardiometabolic risk

The following were used to assess cardiometabolic risk:

- Cardiometabolic index (CMI), created by Wakabayashi and Daimon in 2015, can be an ideal marker for identifying the VAT distribution, and related metabolic dysfunctions. CMI is calculated according to the formula based on TG, HDL, and WHtR values (56). Several studies have shown that CMI is a useful screening tool in various populations, identifying individuals with worsening metabolic abnormalities and a higher CVD risk, such as abnormal left ventricular geometry, arterial stiffness, hyperuricemia, diabetes, hypertension, and ischemic stroke (57–59). CMI consists of anthropometric and biochemical indicators, including waist-to-height ratio (WHtR), and triglyceride to high-density lipoprotein cholesterol (TG/HDL-C) ratio (60, 61),
  - CMI = TG (mmol/L)/HDL-C (mmol/L) × WHtR (62),
  - TG/HDL-C is a simple yet good marker of CVD risk, which closely reflects dyslipidemia and insulin resistance. The TG/HDL-C ratio reflects the presence of small atherogenic LDL-C particles, and is strongly associated with insulin resistance and MetS (63).

### 2.3 Classification of the respondents

The respondents were divided into groups by:

- BMI: where obesity was diagnosed if BMI ≥ 30 kg/m^2^ as recommended by The Centers for Disease Control and Prevention (CDC);
- Smoking: ‘current smoker’ or ‘current non-smoker’;
- Alcohol consumption: ‘teetotaller’ or ‘current alcohol drinker’;
- MetS: ‘no-MetS’ and ‘MetS/pre-MetS’, where MetS was diagnosed according to the International Diabetes Federation (IDF) and the modified National Cholesterol Education Program-Adult Treatment Panel III (NCEP-ATP III) criteria. MetS was diagnosed in women who had at least three of five risk factors, such as (64):
  •WC ≥ 80 cm, TG > 150 mg/dl (1.7 mmol/l) or specific treatment of this lipid abnormality; HDL < 50 mg/dl (1.3 mmol/l) or specific treatment of this lipid abnormality;
  • elevated blood pressure (BP): systolic BP ≥ 130 or diastolic BP ≥ 85 mmHg or treatment of previously diagnosed hypertension;
  • elevated fasting plasma glucose (FPG) level ≥ 100 mg/dl (5.6 mmol/l) or previously diagnosed type 2 diabetes. If it is above 5.6 mmol/l or 100 mg/dl, an oral glucose tolerance test (OGTT) is strongly recommended, but it is not necessary to determine the presence of the syndrome;
  • The women who did not meet the above criteria for MetS diagnosis but had at least two components of MetS were defined as having pre-metabolic syndrome (pre-MetS)
  • WC ≥ 80 cm, TG > 150 mg/dl (1.7 mmol/l) or specific treatment of this lipid abnormality; HDL < 50 mg/dl (1.3 mmol/l) or specific treatment of this lipid abnormality;
  • elevated blood pressure (BP): systolic BP ≥ 130 or diastolic BP ≥ 85 mmHg or treatment of previously diagnosed hypertension;
  • elevated fasting plasma glucose (FPG) level ≥ 100 mg/dl (5.6 mmol/l) or previously diagnosed type 2 diabetes. If it is above 5.6 mmol/l or 100 mg/dl, an oral glucose tolerance test (OGTT) is strongly recommended, but it is not necessary to determine the presence of the syndrome;
  • The women who did not meet the above criteria for MetS diagnosis but had at least two components of MetS were defined as having pre-metabolic syndrome (pre-MetS)
- Menopausal status: the respondents were divided into two groups with regard to their menopusal status (64):
  • perimenopause*―* the time immediately before menopause, when endocrine, biological, and clinical symptoms of impending menopause begin,
  • postmenopause*―*the last menstrual period at least 12 months before the study.
- Abdominal obesity: diagnosed if WC > 80 cm (65).
- HOMA-IR: insulin resistance was diagnosed if HOMA-IR > 2.5 (66).

### 2.4 Statistical analysis

The statistical analysis was performed to explore the associations between various anthropometric and biochemical parameters with circulating levels of adiponectin, visfatin, and adropin. Descriptive statistics were used to summarize baseline characteristics of the study population stratified by BMI categories (normal weight, overweight, and obesity). Continuous variables were reported as means and standard deviations, and comparisons between groups were conducted using one-way ANOVA. *Post hoc* comparisons were applied when necessary.

To investigate relationships between variables, multivariate linear regression analyses were performed. Initially, unadjusted models were used to identify potential associations, followed by adjusted models controlling for confounding factors such as age, menopausal status, smoking, and alcohol consumption. Regression coefficients (β) and corresponding p-values were reported for each model.

Correlations between anthropometric indices (e.g., BMI, VAI, WC, WHtR) and biomarkers were examined both in the general cohort and within subgroups defined by BMI thresholds, smoking status, metabolic syndrome (MetS) presence, insulin resistance (HOMA-IR), and menopausal status. Subgroup-specific analyses employed similar regression frameworks to capture potential effect modifications.

All calculations were performed with the Statistica™ 13.3 software (TIBCO Software, Palo Alto, California, United States). For all analyses, statistical significance was set at p < 0.05.

## 3 Results

### 3.1 Characteristics of the respondents

**Table 1** presents the women’s characteristics and biochemical parameters with regard to their BMIs (according to the categories: normal weight, overweight, and obesity). We found that obese women had significantly higher values of WC (p = 0.041), RFM (p = 0.016), WhTR (p = 0.007), percent body fat *(*PBF) (p < 0.001), and total VAT mass (p < 0.001) than the rest of the subjects, and a slightly lower BSI than their normal-weight and overweight counterparts (p < 0.001). We did not find any statistically significant differences between the groups for other variables.

**Table 1:** General characteristics of the population studied.

Variables	BMI						F	*p*-value*
	Normal weight (n = 46)		Overweight (n = 66)		Obesity (n = 56)			
	M	SD	M	SD	M	SD		
Age	53.65	4.37	54.68	4.62	54.86	5.56	0.878	0.417
Age of menopause*	47.66	4.74	49.22	4.26	48.31	4.53	1.257	0.288
Time since menopause (years)**	8.06	5.65	7.16	4.36	8.64	5.53	0.998	0.372
Glucose [mg/dl]	94.63	37.62	95.23	36.24	89.19	17.74	0.625	0.537
TC [mg/dl]	205.80	36.49	210.89	30.93	204.89	39.73	0.503	0.606
HDL [mg/dl]	64.15	16.24	61.44	16.61	69.09	18.79	3.003	0.052
LDL [mg/dl]	121.15	31.98	126.53	29.38	115.01	35.67	1.927	0.149
TG [mg/dl]	103.73	39.27	118.25	80.15	95.73	43.05	2.233	0.110
CRP [mg/l]	3.51	9.55	2.51	3.26	2.60	3.63	0.466	0.628
HbA1C [%]	5.58	0.74	5.73	1.13	5.45	0.55	1.602	0.205
Insulin [µIU/ml]	10.23	7.01	9.75	5.25	9.90	6.34	0.083	0.920
HOMA-IR	2.43	1.92	2.55	2.67	2.33	2.02	0.153	0.858
IL-beta [pg/ml]	146.19	249.11	148.80	247.64	126.48	238.50	0.142	0.868
TNF-α [pg/ml]	4.46	5.14	4.34	4.52	6.57	11.33	1.538	0.218
IFN-α [IU/ml]	0.14	0.56	0.14	0.33	0.10	0.19	0.174	0.840
IL-6 [pg/ml]	31.19	127.61	38.82	105.88	85.26	180.04	2.373	0.096
IL-1α [pg/ml]	9.76	40.16	4.21	11.23	23.81	110.56	1.319	0.270
Visfatin [ng/ml]	4.57	3.28	5.07	3.88	5.43	4.09	0.649	0.524
Adropin [pg/ml]	448.96	297.99	575.43	425.52	459.47	321.66	2.255	0.108
Adiponectin [ng/ml]	11351.49	6599.47	10528.67	5899.04	11049.16	5913.14	0.263	0.769
SBP	123.00	17.93	123.35	17.69	123.54	19.67	0.011	0.989
DBP	78.22	9.93	78.08	10.00	76.11	12.14	0.667	0.515
WC [cm]	86.14	11.10	90.22	11.58	92.57	15.10	3.248	0.041
Height [cm]	163.96	6.29	163.14	6.47	160.55	7.47	3.695	0.027
Weight [kg]	61.67	5.51	72.40	6.32	89.81	15.17	103.75	<0.001
RFM	37.33	5.02	39.23	5.02	40.44	5.97	4.265	0.016
VAI	1.60	0.95	1.90	1.82	1.16	0.72	4.885	0.009
WhTR	0.53	0.07	0.55	0.07	0.58	0.10	5.146	0.007
LAP	32.92	17.97	41.14	26.70	38.16	24.75	1.605	0.204
*PBF* [%]	37.73	2.06	42.04	1.78	48.69	4.40	177.46	<0.001
VAT	2.95	1.13	3.22	1.19	3.11	1.22	0.671	0.513
Total VAT mass: [kg]	1.84	0.72	2.34	0.88	2.76	1.10	12.528	<0.001
BSI	0.08	0.01	0.08	0.01	0.07	0.01	26.244	<0.001
CMI	0.96	0.59	1.24	1.17	0.89	0.56	2.908	0.057
TG/HDL RATIO	0.92	0.45	0.98	0.71	0.92	0.61	0.190	0.827

**Data for fewer N than indicated in the table header, because not all women were already in the menopausal period.

TC, total cholesterol; HDL, high-density lipoprotein; LDL, low density lipoprotein; TG, triglyceride; CRP, C-reactive protein; HbA1C, glycated hemoglobin; HOMA-IR, homeostasis model assessment of insulin resistance; IL-6, interleukin-6; TNF-α, tumor necrosis factor-alpha; SBP, systolic blood pressure; DBP, diastolic blood pressure; WC, waist circumference; RFM, relative fat mass; VAI, visceral adiposity index; WHtR, aist-to-height ratio; LAP, lipid accumulation product; VAT, visceral adipose tissue; BRI, body roundness index; BSI, body shape index; CMI, cardiometabolic index; M, mean; SD, standard deviation, *N*, whole cohort size; *n*, number of participants in the subgroups, *Student’s t-test, *p*—significance level.

### 3.2 The levels of adiponectin, visfatin, adropin depending on anthropometric parameters

The study analyzed the levels of adiponectin, visfatin, and adropin in the women’s blood in relation to their anthropometric data, taking into account variables such as age, smoking status, menopausal status, and alcohol consumption.

Preliminary multivariate linear regression analysis showed that VAI and LAP were negatively correlated with adiponectin levels (β = -0.428, p < 0.001 and β = -0.428, p < 0.001, respectively). Moreover, LAP was observed to be negatively correlated with CRP (β = -0.310, p < 0.001). Multivariate linear regression analysis adjusted for age and menopausal status demonstrated that VAI was weakly positively correlated with CRP (β = 0.25, p = 0.001) and inversely correlated with adiponectin (β = -0.43, p < 0.001). Similar correlations were noted in preliminary multivariate linear regression analysis adjusted for age, menopausal status, smoking, and alcohol consumption. No other statistically significant correlations were observed between selected anthropometric parameters and the levels of visfatin and adropin (**Table 2**).

**Table 2:** Correlations between obesity indicators and the levels of visfatin, adropin, and adiponectin with regard to age, menopausal status, alcohol consumption, and smoking.

Dependent variable	BMI		WC		RFM		VAI		WHtR		BSI		BRI		LAP	
	β	p	β	p	β	p	β	p	β	p	β	p	β	p	β	p
Log visfatin*	0.105	0.176	0.086	0.266	0.054	0.485	-0.075	0.332	0.072	0.356	-0.012	0.876	0.044	0.572	0.050	0.518
Log visfatin**	0.098	0.209	0.080	0.307	0.048	0.540	-0.080	0.304	0.065	0.404	-0.012	0.881	0.036	0.643	0.041	0.602
Log visfatin***	0.090	0.256	0.078	0.317	0.046	0.553	-0.077	0.324	0.065	0.407	-0.008	0.916	0.019	0.808	0.045	0.566
Log adropin*	0.030	0.700	0.079	0.311	0.048	0.537	-0.038	0.626	0.052	0.503	0.011	0.883	0.043	0.581	0.064	0.410
Log adropin**	0.026	0.736	0.072	0.353	0.041	0.596	-0.040	0.612	0.045	0.569	0.010	0.900	0.034	0.661	0.058	0.456
Log adropin***	-0.009	0.908	0.060	0.440	0.033	0.665	-0.033	0.669	0.038	0.619	0.025	0.748	0.003	0.973	0.058	0.457
Log adiponectin*	0.066	0.396	0.011	0.891	-0.028	0.715	-0.428	<0 .001	-0.004	0.963	-0.040	0.606	0.027	0.725	-0.310	< 0.001
Log adiponectin**	0.066	0.399	0.013	0.871	-0.026	0.737	-0.430	<0.001	-0.001	0.993	-0.039	0.621	0.031	0.696	-0.314	<0.001
Log adiponectin***	0.060	0.458	0.010	0.900	-0.028	0.720	-0.429	<0.001	-0.002	0.980	-0.035	0.653	0.023	0.778	-0.315	<0.001

β, standardized regression coefficient.

*unadjusted regression model.

**regression model adjusted for: age and menopausal status.

***regression model adjusted for: age, menopausal status, smoking, and alcohol consumption.

BMI, body mass index; WC, waist circumference; RFM, relative fat mass; VAI, visceral adiposity index; WHtR, waist-to-height ratio; LAP, lipid accumulation product; BRI, body roundness index; BSI, body shape index.

### 3.3 Correlations of biochemical and anthropometric parameters with the levels of circulating adiponectin, visfatin, and adropin

**Table 3** shows the correlations between anthropometric and biochemical parameters and the levels of circulating adiponectin, visfatin, and adropin.

**Table 3:** Correlations of anthropometric and biochemical parameters with the levels of visfatin, adropin, and adiponectin.

Independent variable	Log visfatin [ng/ml]		Log adropin [pg/ml]		Log adiponectin [ng/ml]	
	β	p	β	p	β	p
Age (years)	0.079	0.309	0.085	0.275	-0.030	0.695
Menopausal age (years)	0.031	0.733	0.085	0.345	0.038	0.674
Body mass (kg)	0.125	0.107	0.054	0.484	0.099	0.202
HbA1C (%)	-0.007	0.933	-0.444	<0.001	-0.444	<0.001
FBG (mg/dl)	-0.032	0.684	-0.322	<0.001	-0.322	<0.001
Insulin (µIU/L)	-0.037	0.633	-0.450	<0.001	-0.450	<0.001
DBP (mm Hg)	-0.041	0.601	-0.087	0.260	-0.015	0.848
Triglycerides (mg/dL)	-0.037	0.637	0.050	0.517	-0.327	<0.001
LDL (mg/dL)	-0.143	0.064	-0.044	0.567	-0.044	0.567
TNF-α (pg/ml)	0.080	0.301	0.033	0.670	-0.024	0.756
IL-6 (pg/ml)	0.154	0.047	0.028	0.722	-0.043	0.581
IL-beta (pg/ml)	0.046	0.554	0.121	0.117	0.055	0.479

HbA1C, glycated hemoglobin; FBG, fasting blood glucose; DBP, diastolic blood pressure; LDL, low-density lipoprotein; TNF-α, tumor necrosis factor-alpha; IL-6, interleukin-6, *p*, significance level.

Preliminary multivariate linear regression analysis confirmed a positive correlation between the levels of circulating visfatin and IL-6 (β = 0.154, p = 0.047). The level of circulating adropin was negatively correlated with HbA1C (β = -0.444, p < 0.001), fasting blood glucose (β = -0.322, p < 0.001), and insulin (β = -0.450, p < 0.001). The level of adiponectin was negatively correlated with HbA1C (β = -0.444, p < 0.001), fasting blood glucose (β = -0.322, p < 0.001), insulin (β = -0.450, p < 0.001), and TG (β = -0.327, p < 0.001).

### 3.4 Correlations of major cardiovascular risk factors with circulating adiponectin, visfatin, and adropin levels

**Table 4** shows correlations between cardiovascular risk factors and the levels of circulating adiponectin, visfatin, and adropin after adjusting for age, smoking, alcohol consumption, and the age of menopause.

**Table 4:** Correlations between cardiovascular risk factors and the levels of circulating adiponectin, visfatin, and adropin after adjusting for age, menopausal status, smoking, and alcohol consumption.

Dependent variable	CRP		HDL		SBP		TC [mg/dL]		HOMA-IR	
	β	p	β	p	β	p	β	p	β	p
Log visfatin*	-0.019	0.809	0.088	0.258	-0.023	0.767	-0.058	0.454	-0.096	0.217
Log visfatin**	-0.017	0.833	0.090	0.255	-0.032	0.681	-0.055	0.483	-0.092	0.237
Log visfatin***	-0.012	0.877	0.076	0.341	-0.033	0.677	-0.067	0.397	-0.091	0.244
Log adropin*	-0.042	0.586	-0.020	0.799	-0.106	0.172	-0.014	0.861	-0.101	0.193
Log adropin**	-0.036	0.646	-0.012	0.875	-0.115	0.142	-0.004	0.964	-0.098	0.206
Log adropin***	-0.021	0.785	-0.041	0.602	-0.108	0.160	-0.026	0.743	-0.093	0.224
Log adiponectin*	-0.124	0.108	0.474	<0.001	-0.092	0.233	0.128	0.097	-0.503	<0.001
Log adiponectin**	-0.130	0.097	0.479	<0.001	-0.092	0.243	0.125	0.113	-0.504	<0.001
Log adiponectin***	-0.127	0.107	0.482	<0.001	-0.091	0.250	0.120	0.131	-0.503	<0.001

β, standardized regression coefficient.

*unadjusted regression model.

**regression model adjusted for: age and menopausal status.

***regression model adjusted for: age, menopausal status, smoking, and alcohol consumption.

CRP, C-reactive protein; HDL, high-density lipoprotein; SBP, systolic blood pressure; TC, total cholesterol; HOMA-IR, homeostasis model assessment of insulin resistance.

Preliminary multivariate linear regression analysis demonstrated that circulating adiponectin levels correlated positively with HDL levels (β = 0.474, p < 0.001), and negatively with HOMA-IR values (β = -0.503, p < 0.001).

Multivariate linear regression analysis adjusted for age and menopausal status showed that adiponectin was positively correlated with HDL (β = 0.479, p < 0.001), and negatively with HOMA-IR (β = -0.504, p < 0.001). Similar correlations were observed in preliminary multivariate linear regression analysis adjusted for age, menopausal status, smoking, and alcohol consumption. No other statistically significant associations were observed between selected variables (CRP, HDL, SBP, TC) and the levels of visfatin or adropin (**Table 4**).

### 3.5 Correlations between anthropometric parameters (BMI, WC, RFM, VAI, WHtR, BSI, BRI, LAP) and the levels of circulating adiponectin, visfatin, and adropin in the subgroups defined by BMI, smoking, MetS, insulin resistance, and menopausal status

The study analyzed correlations between selected anthropometric parameters (BMI, WC, RFM, VAI, WHtR, ABSI, BRI, LAP) and the levels of circulating adiponectin, visfatin, and adropin in the subgroups defined by BMI, smoking, MetS, insulin resistance, and menopausal status (**Supplementary Table S1**, **Table 5**).

**Table 5:** Correlations between anthropometric parameters (BMI, WC, RFM, VAI, WHtR) and the levels of adiponectin, visfatin, and adropin in the subgroups defined by BMI, smoking status, MetS, abdominal obesity, and HOMA-IR.

Dependent variable	BMI < 30.0 kg/m^2^ (n = 112)						BMI ≥ 30.0 kg/m^2^ (n = 55)					
	BMI		VAI		LAP		BMI		VAI		LAP	
	β	p	β	p	β	p	β	p	β	p	β	p
Log visfatin	0.094	0.326	-0.116	0.224	-0.005	0.957	0.070	0.612	0.149	0.277	0.161	0.240
Log adropin	0.188	0.047	-0.115	0.227	-0.007	0.945	0.137	0.317	0.169	0.219	0.204	0.134
Log adiponectin	-0.056	0.556	-0.477	0.000	-0.429	0.000	0.227	0.095	-0.310	0.021	-0.069	0.618
	Current non-smoking (n = 128)						Current smoking (n = 39)					
Log visfatin	0.096	0.282	-0.110	0.218	-0.001	0.994	0.124	0.452	0.078	0.636	0.228	0.162
Log adropin	-0.020	0.826	-0.038	0.667	-0.012	0.891	0.156	0.344	-0.035	0.833	0.253	0.120
Log adiponectin	0.074	0.405	-0.397	0.000	-0.337	0.000	0.060	0.715	-0.573	0.000	-0.249	0.127
	No MetS (n = 78)						Pre-MetS or MetS (n = 89)					
Log visfatin	0.240	0.034	-0.153	0.181	0.046	0.688	-0.061	0.568	-0.103	0.336	0.028	0.795
Log adropin	0.165	0.149	-0.067	0.560	0.150	0.189	-0.151	0.159	-0.029	0.788	0.020	0.854
Log adiponectin	0.100	0.383	-0.197	0.084	-0.040	0.729	0.058	0.588	-0.471	0.000	-0.406	0.000
	Abdominal obesity (n = 128)						No abdominal obesity (n = 39)					
Log visfatin	0.094	0.290	-0.086	0.336	0.053	0.553	0.102	0.536	-0.048	0.772	0.024	0.885
Log adropin	0.011	0.901	-0.079	0.376	0.022	0.803	0.041	0.803	0.058	0.725	0.105	0.525
Log adiponectin	0.130	0.144	-0.544	0.000	-0.366	0.000	-0.088	0.594	-0.066	0.692	-0.010	0.951
	Perimenopause (n = 43)						Postmenopause (n = 124)					
Log visfatin	0.168	0.282	-0.172	0.269	-0.102	0.517	0.068	0.454	-0.017	0.852	0.116	0.198
Log adropin	0.020	0.901	0.038	0.810	0.097	0.538	0.034	0.711	-0.082	0.363	0.052	0.568
Log adiponectin	0.240	0.121	-0.550	0.000	-0.407	0.007	-0.008	0.926	-0.373	0.000	-0.271	0.002
	HOMA-IR < 2.5 (n = 114)						HOMA-IR > 2.5 (n = 53)					
Log visfatin	0.050	0.598	-0.067	0.476	0.006	0.949	0.198	0.155	-0.086	0.538	0.146	0.295
Log adropin	-0.058	0.538	0.034	0.718	0.095	0.317	0.237	0.088	-0.100	0.477	0.060	0.669
Log adiponectin	0.037	0.692	-0.142	0.132	-0.103	0.278	0.177	0.205	-0.515	0.000	-0.381	0.005

BMI, body mass index; WC, waist circumference; RFM, relative fat mass; VAI, visceral adiposity index; WHtR, waist-to-height ratio; BRI, body roundness index; BSI, body shape index; LAP, lipid accumulation product.

In people with BMI < 30.0 kg/m^2^, BMI positively correlated with adropin (β = 0.19, p = 0.047), VAI negatively correlated with adiponectin (β = -0.477, p = 0.000), and LAP negatively correlated with adiponectin (β = -0.429, p = 0.000). In subjects with BMI ≥ 30.0 kg/m^2^, VAI negatively correlated with adiponectin (β = -0.310, p = 0.021). No statistically significant correlations were found between WC, RFM, WHtR, BSI, BRI and the levels of circulating adiponectin, visfatin, and adropin (**Table 5**).

In non-smokers, both VAI and LAP negatively correlated with adiponectin (β = -0.397, p = 0.000 and β = -0.337, p = 0.000, respectively). In smokers, VAI negatively correlated with adiponectin (β = -0.573, p = 0.000). There were no statistically significant correlations between BMI, WC, RFM, WHtR, BSI, BRI and the levels of circulating adiponectin, visfatin, and adropin (**Supplementary Table S1**, **Table 5**).

In subjects without MetS, a positive correlation was observed between BMI and visfatin (β = 0.024, *p* = 0.034). In those with MetS or pre-MetS, VAI and LAP negatively correlated with adiponectin (β = -0.471, *p* = 0.000 and β = -0.406, *p* = 0.000, respectively). There were no other statistically significant correlations between WC, RFM, WHtR, BSI, BRI and the levels of circulating adiponectin, visfatin, and adropin (**Table 5**).

In subjects with abdominal obesity, both VAI and LAP negatively correlated with adiponectin (β = -0.544, *p* = 0.000, and β = -0.366, *p* = 0.000, respectively). No other statistically significant correlations were found between WC, RFM, WHtR, BSI, BRI, and the levels of circulating adiponectin, visfatin, and adropin in the abdominal obesity and no-abdominal obesity groups (**Table 5**). In perimenopausal women, both VAI and LAP negatively correlated with adiponectin (β = -0.55, p = 0.000, and β = -0.407, p = 0.007, respectively). Similarly, in postmenopausal women, both VAI and LAP negatively correlated with adiponectin (β = -0.373, p = 0.000, and β = -0.271, p = 0.002, respectively). No other statistically significant correlations were found between WC, RFM, WHtR, BSI, BRI, and the levels of circulating adiponectin, visfatin, and adropin (**Table 5**).

In subjects with HOMA-IR > 2.5, RFM and WHtR positively correlated with visfatin (β = 0.273, p = 0.048, and β = 0.271, p = 0.045, respectively), while VAI and LAP negatively correlated with adiponectin (β = -0.515, p = 0.000, and β = -0.381, p = 0.005, respectively). No other statistically significant correlations were found between other anthropometric parameters (BMI, WC, BSI, BRI, VAI, LAP) and the levels of circulating adiponectin, visfatin, and adropin (β = 0.271, *p* = 0.045) (**Table 5**).

## 4 Discussion

### 4.1 Correlations of biochemical and anthropometric parameters with the levels of circulating adiponectin

Adiponectin stands out among the three adipokines studied as the most significantly associated with adiposity indices and metabolic serum biomarkers in perimenopausal women, highlighting its unique role in regulating metabolic health. Compared to the other adipokines, adiponectin demonstrates stronger and more consistent associations with metabolic parameters, emphasizing its potential as a key mediator in these processes. Preliminary multivariate linear regression analysis revealed that VAI and LAP were negatively correlated with adiponectin levels. Multivariate linear regression analysis adjusted for age and menopausal status demonstrated that VAI was inversely proportional to adiponectin. Similar connections were found in preliminary multivariate linear regression analysis adjusted for age, menopausal status, smoking, and alcohol consumption. Moreover, the level of adiponectin was negatively correlated with HbA1C, fasting blood glucose, insulin, and TG. Preliminary multivariate linear regression analysis showed that circulating adiponectin positively correlated with HDL, and negatively with HOMA-IR. In multivariate linear regression analysis adjusted for age and menopausal status, adiponectin positively correlated with HDL, and negatively with HOMA-IR. Analogous relationships were observed in preliminary multivariate linear regression analysis adjusted for age, menopausal status, smoking, and alcohol consumption. It is noteworthy that VAI negatively correlated with adiponectin in all respondents after adjusting for BMI, smoking, MetS/pre-MetS, menopause, as well as in women with abdominal obesity or HOMA-IR > 2.5. LAP, on the other hand, negatively correlated with adiponectin in subjects with BMI ≥ 30.0 kg/m^2^, women with MetS or pre-MetS, respondents with abdominal obesity, and those with HOMA-IR > 2.5.

Our findings are largely consistent with existing literature, but several key nuances merit further consideration. The relatively stronger correlations observed between adiponectin and metabolic biomarkers in our cohort may reflect the unique physiological and metabolic dynamics of perimenopausal women, a population characterized by significant fluctuations in adiposity and insulin sensitivity.

Both ours and Kamińska et al.’s findings (67) highlight a negative correlation between visceral adiposity index (VAI) and adiponectin levels. Kamińska et al. confirmed this relationship using multivariate linear regression adjusted for age, menopausal status, and smoking. Similarly, our study demonstrated that VAI was inversely proportional to adiponectin after adjustments for age, menopausal status, BMI, smoking, MetS/pre-MetS, and other factors, with consistent findings in women with abdominal obesity or HOMA-IR > 2.5. Kamińska et al. further identified significant associations of adiponectin with fasting blood glucose, HOMA-IR, and HDL, stratified by menopausal status. Their study showed negative correlations with fasting blood glucose and HOMA-IR and positive correlations with HDL in both premenopausal and postmenopausal women, though the associations extended to insulin in the latter group.

In line with these findings, Abu-Farha et al. (68) identified significant negative correlations between adiponectin and several CVD risk factors, including BMI, WC, systolic blood pressure (SBP), glucose metabolism parameters, HOMA-IR, and triglycerides (TG), while showing a positive correlation with HDL-C and high-sensitivity C-reactive protein (hs-CRP). Similarly, Goropashnaya et al. (69) observed strong associations between adiponectin levels and HDL-C, WC, insulin, and TG, while Jung et al. (70) informed about adiponectin levels being primarily linked to HDL-C, body weight, and HOMA-IR.

Other studies have demonstrated negative correlations between plasma adiponectin levels and cardiovascular risk factors, such as WC and high-sensitivity C-reactive protein (hs-CRP), as well as a positive correlation with HbA1c (71). Wattanapol et al. (72) further showed that serum adiponectin levels negatively correlated with additional CVD risk factors, including WC, body weight, BMI, waist-to-height ratio (WHtR), fasting blood glucose, triglycerides (TG), and LDL-C, while positively correlated with HDL-C. In contrast, Gözüküçük et al. (73) found that adiponectin levels were only negatively correlated with BMI. Von Eynatten et al. (74) reported that circulating adiponectin did not correlate with inflammatory markers (IL-6, CRP, leukocyte count), but was positively correlated with HDL-C and TG, and negatively with the TG/HDL-C ratio. López-Jaramillo et al. (75) emphasized the protective role of adiponectin in cardiometabolic diseases, a finding supported by Calton et al. (76) and Abu-Farha et al. (68), who noted that low adiponectin levels are independently associated with the development of metabolic syndrome (MetS) and hypertension.

Our research results are consistent with those obtained by Christou et al. (77). They claim that adiponectin is down-regulated in states of insulin resistance and cardiovascular disease, and is closely linked to HDL and triglyceride metabolism. Specifically, adiponectin contributes to increased serum HDL levels and enhanced triglyceride catabolism. Moreover, Christou et al. (78) found that patients who experienced a decrease in HDL-C levels within the first month also showed a significant reduction in total adiponectin levels. Conversely, those with increased HDL-C levels over the following two months demonstrated a corresponding rise in adiponectin. Notably, the percentage change in HDL-C after one month was positively correlated with changes in total adiponectin, and over a three-month period, the percentage change in HDL-C was significantly associated with changes in both total and high molecular weight (HMW) adiponectin. In summary, our research, along with other scientists’ studies, has demonstrated a significant correlation between total adiponectin and HDL-C levels, highlighting the interconnected role of adiponectin in lipid metabolism and cardiovascular health.

It is also worth noting that other studies have demonstrated a correlation between adiponectin and various CVD risk factors, further emphasizing its role in cardiovascular health.

The study by Yamamoto et al. (79) not only confirmed the relationship between adiponectin and fasting plasma glucose, insulin, HOMA-IR, total cholesterol, triglycerides, and low-density lipoprotein (LDL)-cholesterol, while also showing a positive correlation with high-density lipoprotein (HDL)-cholesterol, but also highlighted the influence of BMI, systolic, and diastolic blood pressure. Notably, the correlations between serum adiponectin and insulin, HOMA-IR, triglycerides, HDL-cholesterol, and LDL-cholesterol remained significant after adjusting for age, sex, and BMI. Stepwise multiple regression analysis identified HDL-cholesterol, sex, BMI, and HOMA-IR as independent factors significantly associated with serum adiponectin levels.

De Luis et al. (80) also confirmed that adiponectin is negatively correlated with HOMA-IR and positively correlated with HDL-cholesterol. Additionally, serum adiponectin levels are negatively associated with low skeletal muscle mass in obese individuals over the age of 60 with metabolic syndrome. In a study by Baker et al. (81) individuals with exceptionally high adiponectin levels exhibited increased systemic inflammation and a disrupted relationship between adiponectin and the risk of developing diabete.

However, other studies have reported varying findings. Runsewe et al.’s study (82) did not establish serum adiponectin as a reliable surrogate marker for insulin resistance in women with polycystic ovary syndrome, despite the high prevalence of insulin resistance observed in this population. Conversely, He et al. (83) demonstrated that the relationship between abdominal obesity and type 2 diabetes mellitus (T2DM) is mediated by circulating adiponectin levels in adults, suggesting that adiponectin may serve as a potential biomarker for predicting and managing the adverse progression from excess adiposity to T2DM.Wu et al. (84) elucidated that the role of adiponectin in obesity-related hypertension is multifaceted and influenced by the systemic metabolic homeostasis signaling axis. In the context of obesity-induced hypertension, the “adiponectin paradox” emerges from a combination of compensatory mechanisms, adiponectin resistance, and diminished adiponectin clearance due to impaired kidney and liver function. They noted that serum adiponectin levels negatively correlated with VAI.

Gariballa et al. (85) found that increased visceral fat in overweight and obese individuals was correlated with reduced total adiponectin levels. Furthermore, their study revealed that the loss of visceral fat was associated with a significant reduction in inflammatory markers, along with a non-significant increase in total adiponectin levels during follow-up.

In a study by Altinova et al. (86), plasma adiponectin concentrations in overweight individuals were significantly lower compared to those in individuals of normal weight. In overweight subjects, adiponectin levels were negatively correlated with body weight, BMI, systolic blood pressure, fasting insulin, and HOMA-IR, and positively correlated with HDL-C. Overweight individuals with low HDL-C levels exhibited significantly lower plasma adiponectin levels than those with higher HDL-C levels. These findings suggest that circulating adiponectin is associated with insulin resistance and HDL-C levels independently of BMI in overweight individuals.

### 4.2 Correlations of biochemical and anthropometric parameters with the levels of circulating visfatin and adropin

Although our research primarily highlights the significance of adiponectin, it is equally important to acknowledge the roles of visfatin and adropin in metabolic health. A review of the literature shows that there is a relationship between visfatin levels and various types of diabetes: type 1 diabetes (87), type 2 diabetes (88–91), and gestational diabetes (92). According to some researchers, visfatin may play a role in the pathogenesis of diabetes by interacting with the insulin receptor. Others have observed low levels of circulating visfatin in women diagnosed with gestational diabetes (93) and in other forms of diabetes (94). While some studies have reported a positive correlation between visfatin and obesity (95), others noted low plasma visfatin levels in obese patients (96). Furthermore, Ezzati-Mobaser et al. (97) demonstrated a potentially important new role of visfatin in the context of metabolic disorders.

In our study, preliminary multivariate linear regression analysis revealed only a positive correlation between the levels of circulating visfatin and IL-6. No other statistically significant correlations were observed between visfatin and selected variables (CRP, HDL, SBP, TC, anthropometric data). Moreover, in subjects without established MetS, visfatin positively correlated with BMI, while in people with HOMA-IR > 2.5, it positively correlated with RFM and WHtR. No other statistically significant correlations were noted between anthropometric parameters and circulating visfatin levels in the groups after accounting for: BMI, smoking, abdominal obesity, and menopausal status.

In contrast, Zahorska-Markiewicz et al. (98) reported a significant positive correlation between visfatin levels and plasma glucose (but not insulin) in female subjects. Notably, serum insulin concentrations were positively correlated with visfatin levels only in individuals with normal body weight. Olszanecka-Glinianowicz et al. (99) found no association between insulin resistance and visfatin levels. However, in participants without metabolic syndrome (MetS), visfatin levels were positively correlated with triglyceride (TG) levels, insulin, and the HOMA-IR value. Bannigid et al. (100) suggested that serum visfatin levels positively correlated with insulin levels and insulin resistance in women with polycystic ovary syndrome (PCOS), regardless of obesity. According to Alnowihi et al. (101), visfatin levels positively correlated with LDL-C, insulin, insulin resistance, waist and hip circumference, BMI, systolic (SBP) and diastolic blood pressure (DBP), while negatively correlating with HDL-C levels.

Our study revealed a positive correlation between BMI values and adropin levels in individuals with a BMI < 30.0 kg/m². However, no statistically significant correlations were observed between adropin levels and other variables, such as CRP, HDL, SBP, TC, and anthropometric parameters across subgroups defined by BMI, smoking status, abdominal obesity, and menopausal status.

A meta-analysis by Ke et al. (102) indicated that BMI, insulin, glucose, HOMA-IR, total cholesterol, triglycerides, HDL, and LDL may be closely related to adropin levels. Additionally, a significant correlation was found between lower circulating adropin levels and PCOS, with levels significantly lower in PCOS patients compared to healthy controls. Ye et al. (103) demonstrated that adropin levels were negatively correlated with BMI, HOMA-IR, and TG, while positively correlated with HDL. In their linear regression analysis, Erman et al. (104) noted that age, BMI, WC, HDL, fasting glucose, and HOMA-IR were significant factors influencing serum adropin levels. Additionally, adropin levels were negatively correlated with BMI, WC, DBP, insulin, and fasting glucose.

Our study highlights the significant role of adiponectin as a biomarker for lipid profile regulation in metabolic disorders. Given its potential, the assessment of adiponectin levels in plasma may serve as an important prognostic tool for cardiometabolic diseases. However, further research is needed to fully understand the role of adiponectin in the development of cardiometabolic diseases, not only in perimenopausal women but across diverse populations. To substantiate and expand upon our findings, future studies should involve larger sample sizes, longer follow-up periods, and more comprehensive assessments of potential confounding factors. These efforts will provide a deeper understanding of the observed relationships and contribute to advancing the knowledge in this field.

## 5 Potential clinical implications and future research direction

The findings of this study highlight the potential utility of adiponectin as predictive biomarkers for assessing cardiometabolic risk in perimenopausal women. By identifying individuals at heightened risk, healthcare providers can implement early and targeted interventions aimed at mitigating the progression of cardiometabolic diseases. Monitoring the levels of these adipokines could significantly enhance the management of conditions such as diabetes, hypertension, and dyslipidemia, thereby improving overall cardiovascular outcomes. This study also underscores the importance of adopting personalized healthcare approaches tailored to the unique hormonal and metabolic changes experienced during menopause. Implementing such strategies could lead to more effective and individualized treatments, ultimately promoting better health and quality of life for perimenopausal women. Future research should strive to further elucidate the mechanisms underlying the associations between adipokines and cardiometabolic risk. Conducting longitudinal studies is crucial to establish causality and determine the temporal relationship between adipokine levels and the development of cardiometabolic diseases. Additionally, exploring the interactions between these adipokines and other hormonal and metabolic factors across diverse populations could provide a more comprehensive understanding of their roles. Investigating the potential therapeutic targets presented by visfatin, adropin, and adiponectin could pave the way for novel treatment strategies aimed at reducing cardiometabolic risk in menopausal women. Moreover, interventional studies assessing the impact of lifestyle modifications, pharmacological treatments, and dietary interventions on adipokine levels and cardiometabolic health could yield actionable data for clinical practice. These insights could lead to personalized and effective approaches to improving health outcomes for this vulnerable population. By addressing these research areas, we can advance our understanding of adipokines and their significance in cardiometabolic health, ultimately enhancing the quality of care and preventive strategies for menopausal women.

## 6 Limitations and strength

Our study has some limitations. These include a cross-sectional design and a small number of respondents. The research sample consisted mainly of healthy, middle-aged women. Further investigation should involve participants of different ages, with evenly distributed age ranges. It also seems important to conduct research taking into account different ethnic groups, as racial and environmental factors may play a part. Another limitation was the method of insulin resistance assessment because we did not apply the metabolic clamp technique as a reference method of insulin resistance measurement. Consistent with WHO guidelines, menopause was determined by history rather than estrogen levels, with the average time since the last menstrual period being eight years (SD = 5.65). Participants were recruited through information leaflets and posters distributed in public places and advertisements in the local press. However, despite our efforts, our sample may not have been representative.

The strength of this research lies in its focus on postmenopausal women, a high-risk group for cardiometabolic diseases. By providing targeted and relevant findings for this demographic, the study offers a thorough analysis of the relationships between adipokines and various metabolic and cardiometabolic risk factors. This improves our understanding of these complex interactions and contributes valuable insights to the field.

## 7 Conclusions

In conclusion, adiponectin stands out as the adipokine most significantly correlated with obesity indices and metabolic biomarkers in peri-menopausal women, highlighting its key role in regulating metabolic health. Adiponectin may be used or considered as a valuable biomarker for identifying and managing metabolic health in this population. The research findings suggest that adiponectin is not only associated with general adiposity but also with specific markers of metabolic health, such as visceral fat distribution, lipid profile, and insulin resistance.

## Data Availability

The raw data supporting the conclusions of this article will be made available by the authors, without undue reservation.
