# Supplementary material for: Predictive biomarkers for cardiometabolic risk in postmenopausal women: insights into visfatin, adropin, and adiponectin
Source: Front Endocrinol (Lausanne). 2025 Feb 7;16:1527567. doi: 10.3389/fendo.2025.1527567 (PMC11842235; doi:10.3389/fendo.2025.1527567)
Supplement: Supplementary file 1 [file Table1.docx]

**Supplementary Table 1. Correlations between anthropometric parameters (BMI, WC, RFM, VAI, WHtR) and the levels of adiponectin, visfatin, and adropin in the subgroups defined by BMI, smoking status, MetS, abdominal obesity, and HOMA-IR**

|  | BMI < 30.0 kg/m^2^ (n = 112) | | | | | | | | | | BMI ≥ 30.0 kg/m^2^ (n = 55) | | | | | | | | | |
| --- | --- | --- | --- | --- | --- | --- | --- | --- | --- | --- | --- | --- | --- | --- | --- | --- | --- | --- | --- | --- |
|  | WC | | RFM | | WHtR | | BSI | | BRI | | WC | | RFM | | WHtR | | BSI | | BRI | |
|  | β | p | β | p | β | p | β | p | β | p | β | p | β | p | β | p | β | p | β | p |
| Log visfatin | 0.096 | 0.314 | 0.074 | 0.438 | 0.072 | 0.448 | 0.035 | 0.717 | 0.041 | 0.664 | 0.058 | 0.673 | -0.003 | 0.984 | 0.049 | 0.724 | 0.011 | 0.936 | 0.030 | 0.830 |
| Log adropin | 0.076 | 0.426 | 0.039 | 0.684 | 0.044 | 0.647 | -0.040 | 0.674 | 0.067 | 0.482 | 0.123 | 0.371 | 0.108 | 0.430 | 0.115 | 0.402 | -0.007 | 0.959 | 0.016 | 0.906 |
| Log adiponectin | -0.068 | 0.476 | -0.101 | 0.289 | -0.069 | 0.469 | -0.037 | 0.701 | 0.007 | 0.941 | 0.125 | 0.362 | 0.086 | 0.532 | 0.079 | 0.567 | -0.023 | 0.865 | 0.072 | 0.602 |
|  | **Current non-smoking (n = 128)** | | | | | |  |  |  |  | **Current smoking (n = 39)** | | | | | |  |  |  |  |
| Log visfatin | 0.061 | 0.493 | 0.031 | 0.732 | 0.038 | 0.671 | -0.013 | 0.885 | 0.022 | 0.808 | 0.182 | 0.267 | 0.152 | 0.355 | 0.189 | 0.250 | 0.012 | 0.944 | 0.152 | 0.355 |
| Log adropin | -0.016 | 0.862 | -0.014 | 0.878 | -0.032 | 0.721 | 0.007 | 0.942 | 0.014 | 0.871 | 0.289 | 0.075 | 0.230 | 0.159 | 0.260 | 0.110 | 0.040 | 0.808 | 0.185 | 0.260 |
| Log adiponectin | -0.024 | 0.791 | -0.082 | 0.357 | -0.060 | 0.501 | -0.077 | 0.386 | 0.001 | 0.988 | 0.088 | 0.594 | 0.138 | 0.403 | 0.142 | 0.389 | 0.062 | 0.710 | 0.157 | 0.340 |
|  | **No MetS (n = 78)** | | | | | |  |  |  |  | **Pre-MetS or MetS (n = 89)** | | | | | |  |  |  |  |
| Log visfatin | 0.082 | 0.475 | 0.019 | 0.869 | 0.058 | 0.613 | -0.121 | 0.290 | 0.036 | 0.757 | 0.087 | 0.420 | 0.069 | 0.520 | 0.072 | 0.503 | 0.095 | 0.376 | 0.033 | 0.760 |
| Log adropin | 0.173 | 0.129 | 0.113 | 0.323 | 0.135 | 0.240 | -0.032 | 0.783 | 0.077 | 0.505 | -0.029 | 0.788 | -0.014 | 0.893 | -0.032 | 0.763 | 0.069 | 0.521 | 0.031 | 0.770 |
| Log adiponectin | 0.012 | 0.916 | -0.025 | 0.826 | -0.041 | 0.719 | -0.084 | 0.465 | -0.060 | 0.602 | 0.047 | 0.665 | 0.029 | 0.791 | 0.078 | 0.467 | 0.018 | 0.871 | 0.108 | 0.315 |
|  | **Abdominal obesity (n = 128)** | | | | | |  |  |  |  | **No abdominal obesity (n = 39)** | | | | | |  |  |  |  |
| Log visfatin | 0.108 | 0.226 | 0.082 | 0.356 | 0.094 | 0.291 | -0.009 | 0.918 | 0.046 | 0.604 | 0.053 | 0.750 | -0.041 | 0.802 | -0.033 | 0.841 | -0.060 | 0.719 | -0.047 | 0.779 |
| Log adropin | 0.031 | 0.729 | -0.028 | 0.754 | -0.001 | 0.990 | -0.048 | 0.591 | 0.007 | 0.941 | 0.094 | 0.570 | 0.046 | 0.783 | 0.026 | 0.874 | 0.052 | 0.755 | 0.014 | 0.933 |
| Log adiponectin | 0.074 | 0.408 | 0.017 | 0.852 | 0.045 | 0.617 | -0.046 | 0.604 | 0.066 | 0.462 | 0.071 | 0.669 | 0.073 | 0.660 | 0.095 | 0.564 | 0.154 | 0.350 | 0.125 | 0.449 |
|  | **Perimenopause (n = 43)** | | | | | |  |  |  |  | **Postmenopause (n = 124)** | | | | | |  |  |  |  |
| Log visfatin | 0.053 | 0.738 | -0.009 | 0.953 | -0.003 | 0.986 | -0.089 | 0.570 | 0.008 | 0.959 | 0.099 | 0.272 | 0.074 | 0.415 | 0.095 | 0.293 | 0.024 | 0.794 | 0.051 | 0.573 |
| Log adropin | -0.013 | 0.936 | -0.060 | 0.701 | -0.054 | 0.733 | -0.008 | 0.961 | -0.046 | 0.770 | 0.105 | 0.248 | 0.077 | 0.398 | 0.079 | 0.385 | 0.017 | 0.847 | 0.057 | 0.529 |
| Log adiponectin | 0.248 | 0.109 | 0.194 | 0.213 | 0.220 | 0.157 | -0.001 | 0.993 | 0.232 | 0.135 | -0.067 | 0.459 | -0.097 | 0.281 | -0.072 | 0.429 | -0.058 | 0.524 | -0.014 | 0.877 |
|  | **HOMA-IR < 2.5 (n = 114)** | | | | | |  |  |  |  | **HOMA-IR > 2.5 (n = 53)** | | | | | |  |  |  |  |
| Log visfatin | 0.018 | 0.846 | -0.030 | 0.754 | -0.012 | 0.902 | -0.041 | 0.664 | -0.036 | 0.700 | 0.260 | 0.060 | 0.273 | 0.048 | 0.277 | 0.045 | 0.074 | 0.596 | 0.142 | 0.312 |
| Log adropin | 0.067 | 0.478 | 0.030 | 0.753 | 0.032 | 0.738 | 0.047 | 0.618 | -0.027 | 0.777 | 0.113 | 0.421 | 0.110 | 0.433 | 0.115 | 0.411 | -0.069 | 0.624 | 0.149 | 0.287 |
| Log adiponectin | -0.025 | 0.795 | -0.036 | 0.700 | -0.033 | 0.725 | -0.044 | 0.645 | -0.031 | 0.741 | 0.070 | 0.621 | 0.052 | 0.714 | 0.104 | 0.460 | -0.044 | 0.753 | 0.183 | 0.190 |
| BMI—body mass index, WC—waist circumference, RFM—relative fat mass, VAI—visceral adiposity index, WHtR—waist-to-height ratio, BRI—body roundness index, BSI—body shape index, LAP—lipid accumulation product | | | | | | | | | | | | | | | | | | | | |
